# Supplementary material for: Are carbon emissions trading and green financial instruments synergistic? -Comprehensive quantitative research based on content analysis
Source: PLoS One. 2024 Mar 7;19(3):e0298601. doi: 10.1371/journal.pone.0298601 (PMC10919601; doi:10.1371/journal.pone.0298601)
Supplement: S1 Data — (ZIP) [file pone.0298601.s001.zip › Data/地级市Z_F工作报告.zip!/╣π╢1⁄2/╔ε█┌╩╨/└┤╫╘╣1⁄2╓┌║┼í╛┬φ┐╦╩2╛▌═°í┐_2004.docx]

各位代表：

　　现在，我代表深圳市人民政府向大会作工作报告，请予审议，并请各位政协委员和其他列席人员提出意见。

　　一、2003年的工作回顾

　　2003年，在党中央、国务院、省委、省政府和市委的正确领导下，我们以邓小平理论和“三个代表”重要思想为指导，全面贯彻党的十六大和十六届三中全会精神，认真落实胡锦涛总书记、温家宝总理视察深圳的重要指示，按照省委对深圳提出的建设国际化城市的目标定位，把握机遇，扎实工作，克服了非典疫情带来的预想不到的困难，经济建设和社会发展取得了出人意料的好成绩，圆满完成了市三届人大五次会议确定的各项任务。

　　（一）沉着应对，果断决策，夺取了防治非典阶段性重大胜利。

　　面对突如其来的非典疫情，坚决贯彻中央、省和市委的指示精神和工作部署，把防治非典作为当时的头等大事，及时启动应急处理机制，全市人民万众一心、众志成城，广大医护人员恪尽职守、舍生忘死，有效控制了疫情的蔓延，取得了发病人数少、治愈率高、死亡率低、没有一名医护人员感染的好成绩。大力支持科研机构和企业开展抗非典疫苗、药物、试剂、检测仪器的研究开发，在我市及全国的抗非典斗争中发挥了积极作用。加强对外来劳务工的保护，果断处置有关突发事件，保持了社会的稳定。坚持防治非典和经济建设两手抓，及时采取一系列有针对性的应对措施，保障了经济平稳快速发展。

　　（二）经济增长速度加快，综合经济实力和发展后劲进一步增强。

　　经济增长率创8年以来新高。据初步核算，全年实现本市生产总值2860.51亿元，比上年增长17.3%，人均5.39万元，增长7.5%。工业增加值1500.13亿元，增长23.5%。本市生产总值和工业增加值增速为8年来最高。全社会固定资产投资额946.49亿元，增长20.1%。社会消费品零售总额801.77亿元，增长16.3%。地方财政收入337.27亿元，同口径比增长15.6%。

　　实现了“五个1000”的重大突破。外贸进出口总额突破1000亿美元，达到1173.99亿美元，增长34.6%；港口集装箱吞吐量1065.16万标箱，增长39.8%，跃居世界集装箱枢纽港第四位；机场旅客吞吐量1084.28万人次；年度新增工业总产值超过1000亿元；金融机构新增本外币存、贷款余额分别突破1000亿元。

　　三大支柱产业进一步发展壮大。高新技术产品产值2482.79亿元，增长45.2%，占规模以上工业总产值48.93%。自主知识产权产品产值占高新技术产品产值55.85%。软件业实现产值291亿元，出口5.9亿美元，同比增长45%以上。研制成功世界首例获准生产的基因抗癌药物。以海港和空港为龙头的现代物流业蓬勃发展，深圳港货物总吞吐量突破1亿吨，深圳机场货邮行吞吐量40.66万吨。六大物流园区建设加快，物流业信息化水平不断提高，第三方物流服务初具规模，物流企业总数达到2538家。银行、证券、保险等金融产业稳健发展，金融机构本外币存款余额7070亿元，贷款余额5434亿元，分别比年初增长19.5%和23.4%。货币市场、资本市场、外汇市场交易活跃，黄金加工和交易两旺。

　　商贸、旅游、会展等服务业健康发展。市场流通畅旺，重点商贸企业销售额的平均增幅达30%，连锁经营销售额占社会消费品零售总额比重超过40%。以创建“中国最佳旅游城市”为契机，大力开展广深珠旅游联合促销，开通个人港澳游，全年接待游客4284万人次，实现旅游总收入295亿元，旅游外汇收入13亿美元。成功举办第五届高交会、第二届中国（深圳）消费商品采购大会、第八届中国（深圳）国际电机电子零部件采购展及一大批专业展。

　　重大基础设施建设步伐加快。东江水源工程铁石泵站及管道工程建成，北线引水工程按期动工。保供电输变电工程、西部电厂6号机组及一批以大代小机组如期建成投入运行，保障了电力供应。广东液化天然气一期试点工程开工。地铁一期、会展中心、盐田港区集装箱码头三期、蛇口港区集装箱码头二期等一批重大基础设施建设进展顺利。盐坝高速公路B段、107国道宝安段改造竣工通车。完成了贯通特区内外的“一横八纵”城市干线路网规划，若干骨干项目已开工建设。机场老候机楼改造完工投入使用。

　　制定实施一批事关经济长远发展的重大政策。以完善区域科技创新体系为切入点，制定了《关于完善区域创新体系，推动高新技术产业持续快速发展的决定》，提出加快高新技术产业发展的54条具体措施。出台了《支持金融业发展若干规定》及其实施细则，中国国际期货经纪公司等一批重要金融机构迁址深圳或在深设立。制定了《关于支持发展产业集聚基地的若干意见》，服装、钟表、模具、家具、黄金珠宝等产业集聚基地已完成选址和初步规划。出台《关于加快民营经济发展的意见》，全市新增私营企业2.27万户，比上年增长33.6%，民营经济已经成为我市经济的重要组成部分。制定实施了为大企业提供便利“直通车”服务的制度，加大了清费减负力度，切实降低企业营商成本，投资发展环境进一步优化。

　　（三）以国有企业改革为重点的各项改革加快，对外开放迈出新步伐。

　　在国有经济布局战略调整、产权主体多元化、完善国有资产监督管理体制和企业内部管理机制等方面取得新进展。能源集团等5家大型国企完成国际招标招募工作，引进一批境内外战略投资者。出台加快市属企业调整与改组的“1+7”文件，东部集团等14家市属一级国企完成整体改制，机场集团等5家国企全面完成国有资产授权经营，市属上市公司重组工作稳步推进。出台“经营者年薪制实施办法”、“建立长效激励机制的指导意见”和“十条禁令”，建立重大失误追究制度，强化激励与约束机制。市属国有企业资产质量和经济效益进一步改善。

　　文化体制、行政体制、投融资体制等各项改革继续深化。认真落实“文化立市”战略，制定文化体制改革综合试点工作方案，改革文化管理体制，加大公益性文化事业的投入力度，培育文化市场体系。完善深圳报业集团管理运行机制，组建深圳广电集团和发行集团，积极筹备“深圳（国际）文化产业博览会”。继续深化行政审批制度改革，优化审批程序，提高审批效率。推进事业单位改革。交通、环保、教育等领域的投融资体制改革取得新成绩，与香港地铁公司签定了轨道交通4号线建设管理的原则性协议，创造了国内地铁建设新模式。

　　外贸和外资工作不断加强，外源型经济快速增长。外贸出口总额629.62亿美元，增长35.2%。其中高新技术产品出口251.55亿美元，增长60.3%，占全市出口总额的40%。出台《深圳市应对入世行动纲要》，建立反倾销预警机制，积极组织企业应对境外贸易诉讼。全面推进“大通关”工作，口岸通关效率进一步提高。全年实际利用外资50.42亿美元，其中外商直接投资36.23亿美元，增长13.6%。宜家、欧倍德、塔吉特、汤姆逊等世界知名跨国企业来深投资。保税区和大工业区发展态势良好。

　　深港合作走向深入，与内地合作更加密切。皇岗口岸旅检24小时通关顺利实施。深港西部通道工程正式开工，皇岗、罗湖、沙头角等一线口岸改造工程全面展开。抢抓内地与香港更紧密经贸关系安排的机遇，早做工作，3家香港中小银行正式申请在深设立分支机构，一批香港服务业开始登陆深圳，深港两地在服务领域的合作取得新进展。积极参与珠三角城市群规划，参与西部大开发和振兴东北老工业基地建设，扎实开展对口帮扶工作，与国内其他地区的合作更加密切。

　　（四）“净畅宁工程”取得初步成效，城市规划建设管理工作得到加强。

　　大力实施“净畅宁工程”，切实解决群众关心的热点难点问题。认真落实市人大一、二号议案，制定实施标本兼治、责任明晰的“净畅宁工程”实施方案。全面启动了水环境综合整治工作，加大机动车尾气污染防治力度，基本实现了工业污染源达标排放，市容环境有所改善。出台了近期解决交通拥堵问题八项举措，交通组织管理明显加强，在机动车辆大幅增加的情况下，交通不畅的问题有所缓解。完善群防群治体系，深入开展严打整治专项斗争，狠抓打团伙、破大案的工作，强化流动人口、出租屋和娱乐场所管理，加大打击“黄、赌、毒”的力度。完成玉龙坑重大安全隐患整治，加强交通事故预防，安全生产形势平稳，全年未发生重特大恶性安全事故。深入排查矛盾纠纷，重视群众来信来访，落实党政领导接访日制度，及时化解不安定因素，确保社会稳定。

　　按照建设国际化城市的要求，不断加强城市规划建设管理工作。召开全市规划工作南山现场会，强化规划的龙头地位、指导地位和调控地位。编制《深圳市城市近期建设规划》和《深圳市干线道路网规划》，修订《深圳市城市规划标准与准则》。加强城市环境景观和建筑设计，规划深圳湾海滨景观长廊，加大对城市公园的保护力度，通过“国家绿化模范城市”验收和“国家园林城市”复查。严格控制土地开发规模。认真落实市人大“两个规定”，历史遗留违法建筑处理率超过50%。基本完成二线“插花地”行政管理权属移交工作。加快推进宝安、龙岗两区城市化，龙华、龙岗撤镇设街试点工作进展顺利。

　　（五）人民生活进一步改善，各项社会事业全面进步。

　　居民收入和保障水平稳步提高。城镇居民人均可支配收入2.59万元，增长4.0%，农民人均纯收入1.13万元，增长6.2%。居民消费结构升级加快，汽车、住房等大宗商品及休闲度假、保健、文体等服务类消费持续增长。社会保险扩面征缴工作继续加强，基本养老、基本医疗和企业年金参保人数有较大增长。建立了宝安、龙岗两区城市化人员养老和医疗保险制度，两区原村民纳入全市统一社保体系。失业员工保险金、离退休人员养老金100%按时足额发放。完善就业服务体系，努力做好就业和再就业工作，12000多名失业员工实现再就业，城镇居民登记失业率2.6%。打击劳务诈骗和欠薪等违法行为，为劳动者追发工资等1.35亿元。大力落实“民心工程”。继续实施“同富裕工程”，加强困难群众的帮扶工作。降低了法律援助门槛，扩大法律援助覆盖面。住房社会保障体系进一步完善，为60户特困家庭提供了廉租房。

　　教育、文化、卫生、体育等各项社会事业全面发展。全市6区全部建成省教育强区，90%的镇建成教育强镇，增加45所省一级学校，新增8万多个中小学学位，提供1.5万个优质高中学位，多渠道解决非户籍人口接受义务教育问题。高等教育加快发展，深圳大学新增硕士点20个，并开始招收博士生。深圳职业技术学院办学质量稳步提高，深圳信息职业技术学院获国家批准试办示范性软件学院。大学城西校区竣工启用，清华、北大、哈工大正式入城办学，在校全日制研究生近2000人。大力引进海内外各类人才，全年共引进户籍人才3万余人，海外留学生1200多人。文化事业成果丰硕，共获国际级奖项71个，国家级奖项186个。成功举办“深圳读书月”、“大剧院艺术节”、“鹏城金秋社区文化艺术节”等一批大型群众文化活动，取得社会效益和经济效益双丰收。积极推进公共卫生体系建设，完善疾病防控体系。办医体制改革取得突破。新增社区健康服务中心35家。广泛开展全民健身体育活动，成功举办第六届市运会，在第五届全国城运会上金牌数进入前10名。全面稳妥地推行计划生育综合改革，各项指标创建市以来最好成绩。加强国民经济和社会发展监测服务，推行“在地统计”改革初见成效。档案工作信息化水平不断提高。民族宗教事务、外事、对台、侨务等工作取得新成绩。

　　精神文明建设取得新成果。在全社会掀起学习“三个代表”重要思想的新高潮。大力弘扬伟大的抗非典精神，深入学习抗非典英雄和黄联明、熊木春等英模的先进事迹，深圳精神得到进一步弘扬。贯彻《公民道德建设实施纲要》和《深圳市民行为道德规范》，在全社会启动“深圳关爱行动”，城市文明程度进一步提高。实施固本强基工程，基层组织、社区建设得到加强。连续三次被评为“全国双拥模范城”，国防教育、国防后备力量建设继续加强。

　　民主法制建设稳步推进。办理人大议案2件、建议383件和政协建议案3件、提案528件。配合市人大加强立法工作，提请市人大常委会审议法规议案8件，颁布市政府规章5件。提高依法行政水平，进一步完善政府法律顾问制度，严格实行政府重大决策法律论证。增加政府工作的透明度，坚持向市人大报告工作制度，自觉接受政协、民主党派和群众监督，支持鼓励新闻媒体加强舆论监督。主动听取市人大、政协对政府工作的意见和建议，积极支持政协履行职能，加强同各民主党派、工商联、人民团体、无党派人士的联系与合作。

　　（六）狠抓政府自身建设，提质提效提神，办实事求实效抓落实。

　　大力弘扬负责任的精神、自强不息的精神和再创业的精神，提升干部队伍精神状态。强化责任制和协调机制，实行市领导主办工作制度和职能部门主办责任制，理顺部门职责，明确行政责任，提高行政效率。加强行政责任追究和行政效能监察，严肃查处作风涣散、铺张浪费、推诿扯皮、有令不行的行政过失，对几个重大项目进行绩效审计，并鼓励新闻媒体曝光和监督，力求做到“功必赏、过必罚，绩必奖、错必纠”。大力推进反腐保廉工作，积极开展清退违规借贷和逾期借款工作，加大监察、审计部门的监督查处力度。进一步转变政府职能，提高工作标准，降低行政成本。

　　各位代表，过去一年我们能够取得经济社会发展的较好成绩，是全市人民在党中央、国务院、省委、省政府和市委的正确领导下，直面困难，团结一心，奋力拼搏的结果。在此，我代表市人民政府，向奋斗在各行各业的广大干部群众，向中央和各省驻深单位，向驻深解放军、武警官兵和各民主党派、人民团体、社会各界人士及外地来深建设者致以崇高的敬意！向所有参与、关心和支持深圳建设的港澳台同胞、海外侨胞、国际友人表示衷心的感谢！

　　我们清醒地看到，我市经济社会发展还存在一些亟待解决的问题和困难。一是城市发展空间与经济社会发展和人口快速增长的矛盾越来越突出，水、电资源短缺。二是产业结构调整有待加强，第三产业比重持续下降，制造业过于偏向轻型、外向型和加工型，结构性风险较大。三是国有企业改革发展的任务还很艰巨，投融资体制改革相对滞后。四是随着各地招商引资力度加大，深圳吸引外资的竞争优势有所削弱。五是城市规划和管理水平还远不适应国际化城市要求，环境、交通问题仍比较突出。六是社会治安形势依然严峻，人民群众对此反映强烈。七是交通等安全事故时有发生，安全生产隐患仍很严重。八是城市应急反应机制不够完善，应对突发事件的能力有待提高。九是部分政府公务人员的素质、责任心、工作效率和服务水平还不够高、不够强，个别甚至还很差，政府职能需要进一步转变。这些问题，都必须高度重视并在今后的工作中切实加以解决。

　　二、2004年的主要工作

　　市委三届八次全会提出了2004年全市工作的总体要求：以邓小平理论和“三个代表”重要思想为指导，认真贯彻落实党的十六大、十六届三中全会、中央经济工作会议和省委九届四次全会精神，按照建设国际化城市的目标要求，抓住实施内地与香港更紧密经贸关系安排的契机，以发展为主题，以完善社会主义市场经济体制为动力，以人民群众的根本利益为出发点和归宿，坚持能快则快、好中求快，坚持以人为本，全面、协调、可持续发展，团结一心，奋力拼搏，实现经济持续快速协调健康发展和社会全面进步，把深圳的改革开放和现代化建设事业不断推向前进。

　　根据这个总体要求，综合考虑各种因素和各方面意见，建议我市今年的主要经济预期目标定为：本市生产总值比上年增长13%，城镇居民登记失业率控制在3%以内，居民消费价格总指数102%，工业增加值增长17%，高新技术产品产值增长23%，进出口总额及出口总额增长8%，全社会固定资产投资增长15%，社会消费品零售总额增长13%，地方财政收入同口径增长12%。

　　为完成以上目标任务，要着重抓好九个方面的工作：

　　（一）贯彻新发展观要求，加强环境建设，继续推进“净畅宁工程”，提高可持续发展能力和经济社会发展承载力。

　　把贯彻新发展观作为今年政府工作的一条主线。要按照新发展观的要求，转变政府职能，调整政府抓工作的思路。改变传统的重增长、轻保护，先污染、后治理的模式，实现人与自然和谐发展。把财力更多地用于社会管理和公共服务，实现社会全面进步。今年政府直接用于落实新发展观的投资达120亿元，占政府投资计划65%左右，是历年来最高的。今后我们考核各区、各部门的工作，都要把环境指标、社会指标、人文指标与经济增长和引进外资等置于同等重要甚至更为优先的位置。

　　完善城市基础设施建设，努力解决经济社会发展瓶颈问题。水、电等基础设施是城市生存发展的基本要素，是承载经济社会发展的基本平台，要把解决好水、电问题摆在优先位置。大力完善供水水源保障体系，启动东江水源工程二期，建设梅林、笔架山、大鹏支线工程，完成龙口—茜坑供水工程一期，新建扩建一批水库，提高制配水能力。要努力争取南方电网增加深圳电力供应，搞好电源电网建设，加快电厂、输变电设施和以大代小技改工程建设，保障电力供应。推广节能技术，科学用电。加强电力设施保护，确保电力安全生产。结合城市总体规划，完善中长期电力规划。

　　加大环境保护和污染防治力度，净化美化城市环境。进一步落实环保目标责任制，优先保证环保建设资金，加快环境基础设施建设。制定科学合理、切实可行的规划，通过截污、清淤、引水等办法，整体推进“五河四库两湾一口”综合治理，下力气解决河流黑臭问题，确保饮用水源水质达标率。要大手笔研究制定解决我市资源性、景观性缺水的战略方案，认真做好前期论证。继续抓好工业污染防治，提高生活垃圾、生活污水收集处理率。切实搞好污水处理净化后的中水综合利用，大力发展循环经济、清洁生产、绿色消费，提高环境对发展的承载力。加强机动车尾气污染防治，改善大气环境。强化监督检查，进一步整治噪声污染。做好生态城市规划，保障生态安全，大力开展植树造林，启动18个市政及郊野、森林公园建设，力争用3－5年的时间完成。做好城市绿化美化工作，加快城市裸露山体和水土流失治理。发展生态农业、景观农业。高质量建成园博园，办好园博会。高标准做好大鹏半岛的规划设计，使其成为实践新发展观的重要载体。加强市容环境综合整治，狠抓辖区管理责任制，认真解决“城中村”脏乱差问题。坚决制止乱摆卖、乱张贴、乱搭建行为。全力以赴做好“国家环保模范城”和“国家卫生城市”复查的迎检工作。

　　加快交通综合整治和道路建设，缓解城市交通压力。开展“交通教育年”活动，增强全社会交通法规和安全意识。全面落实解决交通拥堵问题的八项措施，进一步提高交通组织和管理水平，加快交通瓶颈改造，打通交通微循环。抓紧“一横八纵”城市干线路网和城市轨道交通建设，确保地铁一期、南坪快速路一期主线工程今年建成通车，实现全市交通状况的整体好转。加快轨道交通1、4号线延长段建设和2、3号线的前期工作，加强东西港区疏港交通规划建设。适度降低中心城区开发强度，合理安排城市功能布局，改善城市空间结构，方便居民就近消费娱乐，缓解中心城区交通压力。建设覆盖全市的公交线网，加快客运枢纽建设，为群众提供安全、舒适的公交服务。尽快建立预防道路交通事故的长效机制，使预防工作责任到人、工作到基层。

　　深入开展社会治安综合治理，确保社会稳定、安宁。大力开展严打整治专项斗争，进一步完善网格化布警，加强路面巡逻，重点打击路面“两抢”等多发性犯罪、严重暴力性犯罪和带黑社会性质的犯罪。加强出租屋和流动人口管理，落实出租屋业主治安责任和案件倒查制度。按照“打防结合，预防为主”的方针，全面加强社会治安防控体系建设，建立单位、企业、社区、村镇领导的治安责任制，完善警民联动的防控网络和群防群治机制。推进公安专业化、公安管理体制和警务运行机制改革，合理设置派出所，加快社区警务建设，强化基层治安管理能力。今年新增3000名警力，充实一线。加强公安队伍建设，坚持从严治警，从优待警，进一步清理整顿保安队伍。建立完善维护社会稳定预警工作机制，加大人民调解工作力度，创新工作方式，培育专业性民间调解组织，及时发现和消除各种不安定因素，高度重视和及时解决群众信访问题，有效防止群体性事件。加强安全生产体系建设，加快调整危险品及油气库布局，消除安全隐患，力争全年不发生重特大安全生产事故。

　　（二）优化结构、提高效益，大力推动产业建设，增创产业发展新优势。

　　加快三大支柱产业发展，把高新技术产业、现代物流业和金融服务业做得更强。抢抓国际产业转移的机遇，完善现有高新技术产业链，加快发展新一代互联网、数字视听、平板显示、集成电路、第三代移动通信等前沿技术和产品，抓住数字多媒体技术主导的影视产品升级与家电信息化的商机，巩固电子信息产业优势。加快生物医药产业、新材料、新能源等新兴产业的发展。进一步完善区域创新体系，整合全市科技经费资源，提高资金使用效益。建设高水平的公共技术平台和高新技术孵化基地。积极支持企业技术创新，重点支持一批科技企业、重大创新项目、重点实验室、工程中心、企业技术中心和科技信息网库建设。加强高新技术产业带的规划、建设和项目引进，加强土地控制，盘活土地存量，分步分片开发。大力实施“厂房再造、产业置换”工程，改造老厂房，主要用于发展软件产业和科技孵化器。

　　大力发展现代物流业。新建成深水泊位5个，新增集装箱吞吐能力130万标箱。加快盐田港区集装箱码头扩建工程、蛇口港区集装箱码头三期工程、大铲湾港区集装箱码头一期工程和铜鼓航道等重大项目的前期准备及建设进度。发展海铁联运，加强与西南等地区的经济互动，增强深圳港辐射功能，进一步提升国际枢纽港地位。推动保税区扩区和港区联动，强化口岸贸易、转口贸易和国际物流服务功能。加快六大物流园区建设，培育一批骨干型第三方物流企业和国际货代市场。继续引进国际国内知名的大企业、大集团来深圳设立采购中心和配送中心。推进“大通关”电子口岸建设，构筑“统一、共享、安全、高效”的物流信息平台，提高物流业信息化水平。

　　积极推动金融产业集聚，促进金融发展。完善金融发展和创新环境，把深圳建设成为金融理念创新、体制创新、产品创新的高地。进一步落实支持金融业发展的18条政策措施，吸引国内外金融机构落户深圳。加强与香港金融业的合作，积极配合香港银行开办个人人民币业务，鼓励支持香港中小银行在深发展。加快推进以资本市场为重点的现代金融服务体系建设，全力推动深交所开设中小企业板。促进货币市场、资本市场、保险市场有机结合，健康发展，强化深圳区域金融中心的地位。配合协助中央驻深金融监管机构加强监管，进一步防范化解金融风险，为建设金融运行安全区服务。

　　积极推动商贸、旅游、会展、中介、信息等服务业发展，提高第三产业在经济结构中的比重。构建“总量平衡、布局合理、辐射力强、活跃繁荣、竞争有序”的现代城市商业网点体系，积极引进现代流通方式，加快华南国际原料城、华润中心万象城等大型商贸项目的建设，加快人民南、华强北等商业片区环境改造，优化宝安、龙岗两区的商业环境，努力建设具有国际水平、代表城市商业形象的标志性商圈。抓好旅游规划和旅游项目建设，整顿规范旅游市场，努力创建“中国最佳旅游城市”。积极推进与香港旅游业的合作，加大国际旅游市场开拓力度，强化深圳作为全国最大的过境游客集散地的地位。以新的会展中心投入使用为契机，全力办好第六届高交会，精心组织培育一批高水平的知名展会，构建与国际衔接、市场化运作的运行模式，逐步形成具有国际竞争力的会展业。培育、规范和加快发展本地中介服务机构和行业协会，积极吸引香港和国内外知名的法律、会计、咨询、经纪、代理、评估等中介机构来深执业和开展合作，全面提升我市专业服务水平。大力发展文化体育产业，培育和完善文化市场，支持音像、影视动画、平面设计业加快发展。

　　发展资本密集、技术密集、清洁环保的装备制造业和基础工业，改造提升传统产业，增创产业发展新优势。充分利用我市区位、市场、经济实力等有利条件，在人力、财力和土地资源等方面采取一些相对倾斜的政策，不失时机地引进一批污染少、能耗低、技术先进、带动作用强的高端装备制造业和基础工业项目，调整、改变我市制造业结构偏轻的状况。抓紧规划建设精细化工基地，有针对性地引进国内外著名的精细化工企业。充分利用我市信息产业发达等优势，力争在机电一体化装备、医疗器械、环保设备等领域取得新的突破。加快用高新技术和先进适用技术改造提升传统产业的步伐，积极引导珠宝、钟表、家具、玩具、服装、印刷、皮革、工艺礼品等行业向加工与设计相结合转变，不断提高设计、工艺水平，提高产品的科技含量、附加值和市场竞争力。继续实施精品名牌带动战略，大力培育深圳本地品牌，加快公共服务平台和产业集聚基地建设。

　　调整优化区域结构，强化“南软北硬”、“南高北重”、“东西两翼旅游港口”的产业分工布局。按照比较优势的原则，自觉主动地强化六个区业已形成的“南软北硬”、“南高北重”、“东西两翼旅游港口”的产业特色。南面特区内的几个区要积极引导、推动产业转移和升级，向二次开发要空间、要效益，在寸土寸金的黄金宝地集中发展金融、物流、高新技术研发、软件、商贸等“软产业”，规划建设新型的都市产业园区，大力发展总部经济、会展经济，体现出高新技术、高端产业和高附加值的“三高”特点。北面宝安、龙岗两区要以“硬产业”为主，加快发展制造业。两区要留足土地，完善政策，抓紧引进一批关乎全市经济发展前途的大项目、基础工业项目，积极承接特区内转移出来的制造业和加工工业。东西两翼利用山海等资源优势集中发展港口经济和旅游经济。

　　（三）围绕建设国际化城市的目标，加强城市功能建设，提升城市综合竞争力。

　　按照国际化城市的功能要求，提高城市的规划建设和管理水平。抓好2030城市发展策略研究，完善城市发展总体规划，做好分区规划和专项规划，提高法定图则覆盖率。全面推行规划的环境影响评价，按照环境评价的要求重新审视、修订既有的规划。全面启动国民经济和社会发展“十一五”规划的编制工作。按照新发展观的要求组织实施城市建设规划，确保拿出更多空间给群众提供公共产品，最大限度地满足人民群众教育、文化、医疗卫生、休闲娱乐的需求。加强城市设计和建筑精品建设，开放和规范建筑设计市场，吸引一流的建筑设计师来深执业。城市发展要从建设为主向建设与管理并重转变，认真学习借鉴香港的城市管理经验，以国际化城市的标准提升城市管理水平。大力整顿土地市场秩序，加强征地拆迁管理和违法违章建筑清理工作，坚决制止新的违法违章建筑产生，积极稳妥地推进“城中村”改造。加大社区建设管理力度，健全公共服务体系，提高社区服务水平。

　　最大限度地增强城市的综合服务和辐射功能。加快国际港口、机场、大型客货场站、口岸等交通枢纽设施和铁路、公路、轨道交通网络的建设，做好机场二跑道的前期规划，大力开拓国际海运、空运航线，形成辐射国内、衔接国际的现代立体交通网络体系。认真做好京广和东南沿海高速铁路客运专线在深圳交汇的筹备工作，努力将我市建设成为重要的铁路枢纽城市。充分发挥区域金融中心的融资平台功能，放大深圳金融产业的服务和辐射作用。不断强化和拓展城市商贸物流功能，进一步增强辐射华南、服务全国的能力。抓住我市36家市属上市公司重组和国内外大企业重组的机会，吸引大产业、大公司来我市发展。创造条件，吸引更多的跨国公司地区总部落户深圳，发展总部经济。

　　加快城市化进程，提升和完善特区外城市功能。兼顾国家、集体和个人利益，加快制定完善土地、户籍、劳动就业、文化教育、医疗卫生、计划生育等配套政策措施，抓好城市化人员养老保险和医疗保险改革，确保城市化的有关工作如期完成。统一特区内外规划建设管理标准，完善两区市政及公共设施投资机制，提高特区外的发展水平，提升城市整体功能。

　　积极推进城市信息化战略，建设“数字深圳”。大力推进以服务公众为核心的电子政务建设，完善部门和专业信息网络，加快基础性战略数据库开发，促进政府信息资源共享，建设全市统一的电子政务网络平台，积极推行网上审批和政府信息网上公开。加快以服务市场主体为核心的电子商务建设，建立行业公共信息服务平台，推进信息技术在企业研发、生产、管理、营销中的应用。加强信息基础设施建设和信息网络安全工作，完善社区信息服务网络，提高社会公共领域和社区信息化水平。

　　（四）按照高品位文化城市的标准，加强城市素质建设，提高城市文明程度。

　　实施“文化立市”战略，加强文化建设，促进对外文化交流。积极稳妥地做好文化体制改革综合试点工作，努力建设文化强市。抓好经营性文化事业单位的转制工作，加快集团化建设步伐，做大做强报业、广电、发行集团。抓紧制定文化发展纲要和配套文化产业政策，鼓励社会力量兴办文化产业和资助文化事业。规划建设一批国际水准的文化基础设施，完成新图书馆、音乐厅、新博物馆工程和大剧院改造，做好歌剧院、现代艺术馆等文化设施的前期准备工作。继续办好“深圳读书月”、“深圳社会科学普及周”等常设文化节庆活动，高水平举办首届“深圳（国际）文化产业博览会”。发挥移民城市优势，积极打造开放包容、兼收并蓄的新时代的城市文化。着力营造文化艺术氛围，聚集多层次文化人才，推动文艺精品生产。丰富和发展广场文化、社区文化等各种形式的文化普及活动，全面提高市民的文化素质。加强对外文化交流与合作，扩大城市知名度。提高外语在公共服务中的普及率，完善英语咨询、导向服务，办好英文报纸、广播和电视节目，提高公务员和市民的外语交往能力。

　　发展教育事业，努力建设教育强市。积极推动教育综合改革试点工作，促进基础教育均衡发展，扩大优质教育资源，抓好教师队伍建设，全面推进素质教育。大力发展高等教育，增强深大、深职院、信息技术学院及民办高校的办学实力和特色。创新办学机制，进一步办好大学城。发展职业教育，强化职业技能培训和劳务工素质教育，鼓励创办社区学院和企业学院，构建终身教育体系，建设学习型城市。牢固树立教育是投资而非开支的理念，继续加大教育投入。建立符合深圳实际的多元教育投资办学体制，鼓励发展和规范管理民办教育。加快引进国外著名高校合作办学，加强深港教育合作与交流，继续办好国际学校和港澳台胞子弟学校，提升教育国际化水平。大力推进科普事业，提高市民的现代文明意识和科学素质。

　　大力实施人才战略，建设人才高地。认真落实全国人才工作会议精神，树立人才是第一资源的观念和科学的人才观，坚持培养、引进和使用并重，完善育才、引才、聚才和用才机制，建立科学的人才评价、使用机制，为各类人才脱颖而出创造良好的环境，促使深圳成为全国精英人才汇聚的高地。继续加强企业博士后工作站建设，办好留学生创业园。充分发挥深圳机制灵活、创新活跃、环境较好的优势，发挥用人单位引进、培养、使用人才的主体作用，大力引进海内外高素质人才、创业型人才和适应国际化城市需要的人才，建设一支高素质的专业人才队伍和以高级技工、技师为骨干的产业工人队伍。

　　积极推进“诚信深圳”建设，整顿规范市场经济秩序。建设诚信政府，发挥政府在诚信建设中的表率作用。加强诚信道德教育，形成以道德为支撑、产权为基础、法律为保障的社会信用制度，降低城市经济社会活动交易成本。进一步完善企业和个人征信系统，建立信用监督和失信惩戒制度，开展“守信企业”创建活动。整顿规范市场秩序，坚决打击偷、逃、骗税、恶意逃废金融债务、制假售假、走私贩私、不正当竞争、传销和变相传销等违法行为。加强部门联动配合，争取用一年左右时间基本解决无证无照经营问题。大力整治非法诊所。全面实施食品药品放心工程，加强食品安全和药品、医疗器械监督检查，提高农产品的质量安全水平。加强知识产权保护，严厉打击各类侵犯知识产权的行为。

　　（五）以改革为动力，促进多种所有制经济共同发展。

　　大力发扬改革精神，为发展注入强大动力。改革创新是深圳特区的标志、优势和灵魂，是下一步继续发展的动力、继续领先的法宝，是深圳发展永恒的主题。特区之特，最重要的经验就是敢闯敢干、勇于创新，这种精神永远都不能丢。改革问题关系到深圳的发展后劲和出路，在新形势下我们更要继续全力推进各项改革，向改革要空间、要潜力、要动力，发扬改革的精神，加大改革的力度，拓宽、深化改革的路子，书写改革的新篇章。

　　积极推进国资管理体制和国有企业改革。坚持政府公共管理职能和国有资产出资人职能分开，积极探索创建既符合国家总体要求，又有深圳特色的国有资产管理和监督体制。剥离机关事业单位经营性国有资产，统一纳入国有资产监管和营运体系，加强对非经营性国有资产的清理和监管。以产权改革为核心，大力推进国有企业改革，发展混合所有制经济，加快实现国有企业产权主体的多元化和社会化，使股份制成为公有制的主要实现形式。推动更多的国有企业股权向国内外有实力的公司招标招募。继续试行管理层收购产权和员工持股等形式的企业整体改制，积极探索国有企业管理层和员工在产权层面与企业形成命运共同体的机制。认真做好竞争性领域劣势国有企业的有序快捷安全退出工作。加大市属国有上市公司重组力度，整合现有资源，培育做大一批具有国际竞争力的企业集团。按照现代企业制度的要求，完善公司法人治理结构，落实企业领导人员聘任制度，改进出资人对经营管理者的考核评价体系。切实执行“十条禁令”，进一步完善国有企业经营者的监督和约束机制。以明晰产权为重点深化集体企业改革，发展多种形式的集体经济。加强对股份合作公司的管理，引导其按照现代企业制度进行改制，规范发展。

　　大力扶持民营经济和中小企业发展。全面贯彻实施《中小企业促进法》，落实《关于加快民营经济发展的意见》，清理和废止限制民营经济发展的行政规章和政策，放宽市场准入，鼓励非公有资本进入法律法规未禁入的基础设施、公用事业及其他行业和领域，为民营经济和中小企业营造公平、宽松的创业发展环境。落实民营及中小企业发展专项资金，完善以信用担保为重点的服务体系建设。实施“民营企业50强”计划，支持民营及中小企业进行管理创新、技术创新和机制创新，提高民营及中小企业的整体素质和核心竞争力。

　　（六）加大固定资产投资力度，实现投融资体制改革的新突破。

　　力促固定资产投资再上新台阶。今年计划安排重大建设项目205个，总投资2330亿元，年度投资238亿元，比上年增长19%。要继续完善政府投资项目分类指导体系，条件成熟的项目不受年度投资计划、投资规模和资金安排的限制，成熟一个上一个，充分发挥投资对经济的拉动作用。积极扩大投资项目储备库，重点在“净畅宁工程”和枢纽型、功能性、网络化基础设施，以及产业结构调整优化方面挖掘项目源。加快光明高新技术产业园区、大工业区、保税区新扩区域的开发建设和重大项目引进，适应宝安、龙岗城市化需要，加大两区水、电、路和环保等基础设施建设力度。

　　全力推进投融资体制改革。加快以市场为导向的投融资体制改革，是满足我市国际化城市建设需要，力促固定资产投资再上新台阶的重要保障。要在总结以往经验的基础上，抓紧制定政府投资项目融资整体方案，今年务必要在投融资改革方面取得更大的突破，显著提升政府投资对社会投资的杠杆作用和放大效应。切实推进投资主体多元化和融资方式多样化，进一步缩小政府财政投资的范围和领域，把财政资金挤占的信用空间让出来，充分发挥金融资本、社会资本的作用，努力改变我市银行业存贷比过高的现状。全力推进交通、环保、水务、高新技术产业带、区镇城市化及社会事业等领域的投融资体制改革。推广引进香港地铁公司参与轨道交通4号线建设和南开深圳金融工程学院利用社会资金建设大学的经验，积极探索利用多种融资形式，多渠道筹集建设资金。充分利用资本市场，切实用好世界银行、国家开发银行等的中长期贷款，积极争取开展发行地方政府建设债券、设立产业投资基金、改革社保基金运营方式等试点工作。探索政府项目建设的市场化新路子，将项目融资与项目建设结合起来。加快组建基础设施投资公司。加强政府投资项目的科学论证和前期准备工作，建立重大投资项目公示制度，完善全过程跟踪审计监督，确保政府投资效益最大化。

　　（七）扩大对内对外开放，加强区域经济合作。

　　大力发展开放型经济，提高招商引资的质量和水平。改变“坐商”、“等商”的传统意识和做法，主动出击，切实推动引进外资方式的转变。通过产业链招商、园区整体招商、以商引商、代理招商等方式，大力吸引国内外各类企业投资深圳。以产业结构调整为导向，引进大规模集成电路制造、整机制造和软件等创新型高科技产业，实现汽车和汽车电子及零配件、精细化工等行业引进内外资的突破。着力吸引跨国公司把核心制造环节、研发机构和采购中心转移到深圳，进一步吸引、扩大世界500强在深投资。积极引导加工贸易优化升级和落地生根，向深度加工、研发、营销等复合型功能转变。

　　积极应对出口退税政策的调整，继续保持外贸出口的增长态势。凡是在深圳投资生产并出口的企业，要百分之百、一分不少地确保地方承担的退税得到落实。坚持以质取胜和市场多元化战略，提高出口商品的技术含量和附加值，扩大海外销售网络，巩固欧美等发达国家市场，拓展东盟、中东、独联体、东欧和非洲等地区市场。加强对世贸知识的学习、研究和培训，有效应对国际贸易摩擦，组织好本地企业的反倾销应诉工作。积极支持有条件的企业“走出去”，到国外设立研发中心、生产基地、销售网络，开展跨国经营，带动产品、技术和劳务出口。进一步完善对外投资服务体系，加强海外推介活动，培育一批深圳的跨国企业和著名品牌。

　　紧紧抓住内地与香港更紧密经贸关系安排实施的机遇，在粤港合作的框架下，进一步加强深港合作。积极推进建立双方领导人的定期磋商机制，加强深港政府间的沟通协商，鼓励和支持民间交流。密切与香港在口岸管理方面的合作，进一步整治口岸通关环境，促进通关便利化。加快推进西部通道、铜鼓航道、一线口岸改造等跨境基础设施工程，推进两地轨道交通网的接驳，实现两地城市功能的顺利对接。加大城市基础设施领域对港资开放，做好与香港地铁公司合作建设轨道交通4号线工作。积极吸引和承接香港服务业，组织好双向互动式的招商推介活动，拓展两地在金融、物流、商贸、会展、旅游、专业服务等领域的合作，促进我市现代服务业迈向更高水平。加强两地在高科技领域的合作，共同商讨河套地区的开发建设问题。

　　大力推进区域经济合作，努力开拓国内市场。加强与大珠三角各地区的合作，做好规划、产业、基础设施等方面的衔接，共同构筑一个分工合作、相依相通、共存共荣的区域经济体系。积极融入泛珠三角经济合作，增强深圳作为内地各省区引进外资和对外经济联系的出海通道和窗口作用。继续组织企业参加各地举办的商贸洽谈会，完善内销网络，提高国内市场占有率。积极参与西部大开发和东北老工业基地振兴，鼓励我市企业到中西部地区和东北地区，结合开拓市场投资办厂、兼并收购企业、拓展服务业，形成以深圳为基地、辐射全国的生产和营销服务体系。积极做好对口扶贫工作，创新扶贫工作的机制和方法，完成好国务院和省政府下达的对口帮扶任务。

　　（八）加强社会主义民主法制和精神文明建设，推动各项社会事业全面进步。

　　扎实推进民主法制建设。政府的一切权力都是人民赋予的，必须对人民负责，为人民谋利，自觉接受人民的监督，在监督下开展工作，在阳光下运用权力。有了人民监督，政府才不会懈怠，才能保持高效。各级政府要坚持向人大及其常委会报告工作制度，充分尊重和发挥人民政协、各民主党派、人民团体在政治协商、民主监督和参政议政中的作用，切实做好人大、政协各项议案、建议、提案的办理工作，充分发挥新闻媒体的舆论监督作用。大力推进政务公开法定化，增强政府工作透明度。改革和完善行政决策机制，提高决策的公众参与程度，推进决策的科学化、民主化和程序化。配合市人大做好立法工作。坚持依法行政，按照政府法定权限和程序行使权力、履行职责，善于依法处理经济社会事务，严格执法、公正执法、文明执法。落实行政执法责任制和过错追究制，强化行政复议的层级监督职能，认真对待行政诉讼。改进法律服务，支持仲裁事业发展。加大普法力度，提高公民法律素质。继续加强社区基层组织建设和民主法制建设。

　　切实加强精神文明建设。加强邓小平理论和“三个代表”重要思想的学习教育，弘扬“开拓创新、诚信守法、务实高效、团结奉献”的深圳精神和民族传统美德，增强广大干部群众热爱深圳、热爱家园、关心社会的责任感、归属感，提高城市凝聚力。贯彻《公民道德建设实施纲要》和《深圳市民行为道德规范》，进一步提高市民的思想道德水平。深入实施“深圳关爱行动”，推进“绿色社区”、“青年文明号”、“巾帼文明示范岗”等创建活动和志愿者行动，培育良好的社会风尚，力争深圳进入全国首批文明城市行列。

　　大力发展各项社会事业。加大社会事业投入，促进经济社会协调发展。加强基础研究和应用研究，实施专利战略、标准战略，促进科技与经济的紧密结合。繁荣发展哲学社会科学。按照中央和省的统一部署，坚持疫情防治和经济建设两手抓，防非典、防高致病性禽流感和发展、稳定三兼顾，加强公共卫生体系建设，落实防控工作责任制，提高应对突发性公共卫生事件的能力。抓紧建设新的疾病预防控制中心和传染病医院，改造急救中心，新建、改扩建一批专门和综合医院。深化医疗卫生体制改革。深入开展爱国卫生运动，加强健康教育，广泛开展全民健身运动。加快公共体育设施规划建设，做好6—8万人大型体育场馆项目的前期研究论证。抓紧备战第十二届省运会，争创竞技体育新佳绩。制定科学的人口政策，优化人口结构，提高人口整体素质。抓好计划生育工作，强化流动人口的计生管理和服务，全面落实计划生育优待优惠政策。加强统计工作，按全国统一部署搞好第一次经济普查。做好征兵、民兵预备役、双拥共建和优抚安置工作，提高市民国防意识，巩固发展军政、军民团结。重视民族宗教事务，发挥外事、侨务、港澳台务等在对外交往和扩大开放中的积极作用，进一步加强边防、档案、保密等各项工作。

　　不断改善人民群众生活。落实“十项民心工程”。实施就业优先战略，完善市、区、街道（镇）三级就业服务网络，强化就业和再就业技能培训，加强职介机构管理，积极开发公益性岗位，扩大再就业。完善社会保障制度，扩大社保覆盖面，加强社保基金管理。关心困难群众，帮助他们解决生活、就业、医疗、住房、子女就学等方面的实际困难。及时发现和解决实施救助管理体制中出现的新问题。健全完善劳动安全、劳动仲裁和法律援助制度，加强劳动关系协调与劳动监察，切实保障劳动者合法权益。立法解决欠薪问题，坚决清退拖欠的工程款项和劳务工工资。积极支持老龄工作和残疾人事业，切实保障妇女和未成年人合法权益。继续实施“同富裕工程”，促进我市欠发达地区早日脱贫致富。

　　（九）求真务实，努力建设负责任、有权威、廉洁高效的服务型政府。

　　办实事求实效抓落实，切实履行政府职责。抓落实是政府工作的职能定位，是政府的首要职责，是实现今年工作目标，完成各项任务的前提、基础和关键。对市委三届八次全会和本次人大会议确定的工作思路和部署，市政府就是要在抓落实的行动上下功夫、做文章，讷于言而敏于行。要大力弘扬负责任的精神，真抓实干，言而有信，知难而进。要按照新修订的《深圳市人民政府工作规则》的要求，进一步理顺政府工作流程。强化职能部门的主办责任制和抓落实的工作机制。要坚持正确的政绩观，顾全大局，统筹兼顾，立足当前，着眼长远，不急功近利，绝不搞短期行为，一切从实际出发，按客观规律办事，使我们的工作经得起实践、历史和群众的考验。要深入基层，深入实际，深入群众，反对文牍主义，反对形式主义。完善经济运行监测和预警体系，提高市场经济条件下政府驾驭经济工作的能力。把抓落实的能力和负责任的精神作为考核公务员和政府部门工作的重要标准，将政绩考评与群众公议挂钩，使各级政府真正做负责任的政府，全体公务员真正做负责任的公务员。

　　大力整饬政风，确保政令畅通。公务员队伍素质决定政府管理水平和效率，代表政府的形象。要大力加强政风建设和公务员队伍建设，从严治政，进一步整顿机关作风，戒除骄惰散漫之气，从领导干部入手，从细微之处入手，推动公务员队伍提质提效提神，建设一支政治坚定、业务精通、作风优良、纪律严明、恪尽职守、能打硬仗、善于服务、甘于奉献的公务员队伍。要敢抓敢管，敢于碰硬，敢负责任，真正树立政府权威。要想人民群众所想，急人民群众所急，不畏压力，不徇私情，不迁就，不推诿，有一种一抓到底，不达目的不罢休的胆识和魄力。对有令不行、有禁不止、行政不作为，或超越法定权限、违背法定程序行事，在群众中造成恶劣影响的部门、单位和个人，要坚决追究当事人及直接领导的责任，决不手软。行政监察部门要切实加大监督检查力度，对于违纪部门和责任人，不论涉及到谁，都要一查到底，绝不姑息，使我市政风有显著改善。

　　贯彻落实《行政许可法》，大力推进行政体制改革创新，全面建设服务型政府。

　　今年7月1日《行政许可法》将正式施行，这是规范政府行为的一部重要法律，其所确立的一系列行政许可制度，对进一步规范政府行为，提升政府依法行政的能力和水平，形成与社会主义市场经济体制相适应的新的行政管理机制和管理方式，都具有重大而深远的意义。各级政府和每个公务员都必须认真学习、准确理解、熟练掌握行政许可法的各项规定，树立设权应法定、有权必有责、用权受监督、侵权须赔偿的法治观念。要抓紧做好对行政许可规定和实施机关的清理工作，改革行政许可实施的方式。结合行政许可法的实施，加快第三轮行政审批制度改革，认真组织实施省委、省政府批准的行政管理体制改革方案，加快机构职能调整，实现政府职责、机构和编制的法定化，强化政府经济调控、市场监管、社会管理和公共服务的职能，建立有限政府、服务型政府。要牢固树立“群众是主人，公务员是公仆”、“企业就是顾客，客户至上”的理念，正确处理依法规范行政和优质高效服务的关系，主动围绕市民与企业的需求开展工作，以方便市场主体为导向调整安排工作程序，借鉴现代企业的运作理念，不断推出新的服务产品和服务方式。在完善大企业“直通车”服务的基础上，进一步建立政府职能部门与企业间定期沟通机制。

　　艰苦奋斗，厉行节约，建设清正廉洁、高效低耗的政府。要本着为纳税人负责的态度，精打细算，厉行节约，努力提高财政资金的使用效益，严厉查处把政府财政当“唐僧肉”，狮子大开口，大手大脚，不负责任的行为。继续深入开展清收各类违规和逾期借款工作。加大政府财力的整合力度，分步将政府性基金纳入到国库集中支付的范围，集中财力办大事。进一步完善政府采购制度，加强部门预算管理，逐步实现部门预算与综合业绩挂钩，硬化预算约束。坚持依法独立审计，强化财政资金的绩效审计，扩大绩效审计范围，完善责任追究制度。降低政府运作成本，将财力更多地投向公共服务和公共产品。要大力加强廉政建设和反腐败斗争，坚决查处违纪违法案件，“廉”字当头，干净干事，廉洁从政。

　　各位代表，深圳的发展已处于一个较高的平台上，现在上上下下对我们期望很高，方方面面对我们特别关注，全国各地都在加快发展，已成百舸争流的态势，竞争十分激烈，不进则退，慢进亦退。我们面临的挑战是严峻的，压力是巨大的，我们只能自强不息，顽强拼搏，自己不倒谁也打不倒，自己不垮谁也搞不垮，自己不沉沦谁也不能使我们沉沦！

　　各位代表，深圳是一个干事创业的好地方，是一个能干事、能干大事、能干成大事的地方，我们具备很多进一步开拓发展的有利条件和优势。我们既要有忧患意识、危机意识，更要有自信心，要有一种大气、豪气，有一种藐视困难、战胜困难的勇气。只要我们继续保持改革开放之初的那种敢闯敢试、敢为天下先的精神，保持谦虚谨慎、不骄不躁的作风，心齐劲足实干，我们的目标就一定能够实现。让我们高举邓小平理论伟大旗帜，努力实践“三个代表”重要思想，全面贯彻党的十六大和十六届三中全会精神，在党中央、国务院、省委、省政府和市委的正确领导下，心稳神定，心无旁骛，努力完成全年各项工作，为率先基本实现现代化，建设国际化城市的宏伟目标而努力奋斗！
